# Supplementary material for: Human recombinant erythropoietin improves motor function in rats with spinal cord compression-induced cervical myelopathy
Source: PLoS One. 2019 Dec 10;14(12):e0214351. doi: 10.1371/journal.pone.0214351 (PMC6903714; doi:10.1371/journal.pone.0214351)

***PLoS One* Supporting Information**

**S1 Fig**

Article title: Human recombinant erythropoietin improves motor function in rats with spinal cord compression-induced cervical myelopathy

Authors: Tanaka T, Murata H, Miyazaki R, Yoshizumi T, Sato M, Ohtake M, Tateishi K, Kim P, Yamamoto T

The following Supporting Information is available for this article:

S1 Fig. Time course of body weight

Although body weight was affected after surgery, as evident from the reduction in weight gain at 1 week, no significant difference was observed between the groups. The body weight gain was equal among all groups thereafter, and no significant difference was observed at any week.


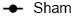

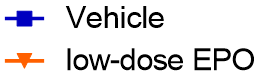

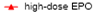

Supplement: S1 Fig — (DOCX) [file pone.0214351.s001.docx]
